# Acute Promyelocytic Leukemia With Long and Short Isoforms of PML::RARA Fusion Transcripts Complicated by Abdominal Distension and Acute Edematous Pancreatitis During Induction Treatment: A Case Report

**DOI:** 10.7759/cureus.60321

**Published:** 2024-05-15

**Authors:** Songlin Chu, Jun Bai, Xin Wang, Liansheng Zhang, Lijuan Li

**Affiliations:** 1 Department of Hematology, The Second Hospital and Clinical Medical School, Lanzhou University, Lanzhou, CHN

**Keywords:** acute edematous pancreatitis, abdominal distension, differentiation syndrome, mixed fusion transcripts, acute promyelocytic leukemia

## Abstract

The introduction of all-trans retinoic acid (ATRA) and arsenic trioxide (ATO) has transformed the outcome of acute promyelocytic leukemia (APL) from a uniformly fatal disease to one of the most curable human malignancies in recent decades. However, early mortality caused by coagulopathy, infection, multi-organ failure, and differentiation syndrome (DS) during disease onset and induction treatment remains a major issue in APL, especially in elderly patients who may suffer from higher treatment-related mortality due to a higher vulnerability to treatment toxicities. Herein, we present a case of an elderly patient with APL with rare mixed long (L-) and short (S-) isoforms of PML::RARA fusion transcripts who had multiple complications at disease onset. In addition, the initiation of treatment with ATRA in combination with ATO led to the rapid onset of severe DS. In particular, this patient experienced a rare clinical feature of DS, acute edematous pancreatitis (AEP). Furthermore, due to the patient's refractory abdominal distension related to the dose of ATRA, ATO, and Realgar-Indigo Naturalis Formula (RIF), we have to repeatedly adjust the doses of these drugs that the patient can maximally tolerate. Nevertheless, the patient achieved complete remission (CR) even after receiving a substandard dose of these drugs. However, the patient relapsed, acquired the FLT3-ITD mutation nine months later, and experienced abdominal distension again while receiving the standard doses of ATRA and RIF. Therefore, these drugs were adjusted to the maximum tolerated dose based on the experience with the initial induction treatment, and the patient achieved CR after four weeks of reinduction treatment. We report that this case may provide some clinical information for the diagnosis and treatment of similar patients with APL.

## Introduction

Acute promyelocytic leukemia (APL) is a biologically and clinically distinct subtype of acute myeloid leukemia (AML) characterized by the PML::RARA rearrangement resulting from the t(15;17)(q24;q21) translocation. According to the different breakpoints of the promyelocytic leukemia (PML) gene on chromosome 15, PML::RARA fusion transcripts can be divided into three isoforms known as L-, variant (V-), and S-isoforms, respectively. However, less than 2% have some other partner genes with the retinoic acid receptor alpha (RARA) gene on chromosome 17, such as ZBTB16, NPM1, STAT5B, IRF2BP2, etc. [1]. The sub-isoforms of APL are associated with heterogeneity in terms of clinical features, hematological parameters, treatment response, and prognosis. For example, the V-isoform has a relatively low sensitivity to all-trans retinoic acid (ATRA); the V- and S-isoforms frequently have higher initial leukocyte counts and correspondingly higher risk compared to the L-isoform; the S-isoform is prone to developing DS during induction treatment and also more likely to acquire additional chromosomal aberrations that may lead to a poorer prognosis [2]. However, risk stratification and the development of treatment protocols for APL have been based primarily on initial leukocyte and platelet levels. Whether the sub-isoforms of PML::RARA have any prognostic value remains to be determined.

DS is a major cause of early death in APL. Therefore, it is critical to recognize the laboratory indices and clinical symptoms of DS and intervene promptly. DS often occurs when the WBC is higher than 10 x 10^9^/L and continues to rise during ATRA and/or arsenic trioxide (ATO) treatment, but it can also occur when the white blood cell (WBC) count is relatively low. The current diagnosis and severity assessment of DS is based on seven clinical signs, including unexplained fever, dyspnea, pleural or pericardial effusion, pulmonary infiltrates, renal insufficiency, unexplained hypotension, and weight gain >5 kg. It is noteworthy that weight gain indicates progression to DS, and dyspnea is the predominant clinical feature in early DS. In addition, DS may have other rare manifestations, such as diffuse alveolar hemorrhage or acute febrile neutrophilic dermatosis [3]. Acute pancreatitis (AP) is a rare complication of APL, most commonly related to hypertriglyceridemia from ATRA treatment. However, a small percentage of APL patients develop AP in the absence of hypertriglyceridemia [4]. AP as a symptom of acute arsenic toxicity is much rarer and always associated with other clinical manifestations such as diarrhea, abdominal pain, and jaundice. Therefore, the possible causes of APL associated with AP need to be further summarized.

## Case presentation

A 70-year-old man was admitted to the hospital with a one-month history of fatigue and a two-day history of buccal mucosal purpura. We define the first day of admission as day 1 (D1). Table 1 shows the patient's most important test results at the time of initial admission.

**Table 1:** The patient's main test results at the time of initial admission and at the time of relapse nine months later. ^*^PML::RARA was tested qualitatively by fluorescence PCR capillary electrophoresis fragment analysis and quantitatively by quantitative real-time PCR. WBC, white blood cell; Hb, hemoglobin; PLT, platelet; PT, prothrombin time; APTT, activated partial thromboplastin time; FIB, fibrinogen; FDP, fibrinogen degradation products; urea, urea nitrogen; CREA, creatinine; UA, uric acid; LDH, lactate dehydrogenase; GLU, blood glucose; BM, bone marrow; CD, cluster of differentiation; PCR, polymerase chain reaction

Test items (normal range)	Initial admission	Relapse nine months later
WBC (3.5-9.5 × 10^9^/L)	6.8	5.9
Hb (130-175 g/L)	91	128
PLT (125-350 × 10^9^/L)	20	22
PT (9.4-12.5 seconds)	13.2	12.6
APTT (25.4-38.4 seconds)	28.6	29.2
FIB (2.00-5.00 g/L)	1.77	1.60
D-dimer (﹤0.50 μg/mL)	35.00	8.32
FDP (﹤5.00 μg/mL)	98.01	45.91
Urea (3.60-9.50 mmol/L)	12.74	9.70
CREA (57-111 μmol/L)	104.90	126.90
UA (208-428 μmol/L)	465.00	448.10
LDH (120-250 U/L)	1249	614
GLU (3.90-6.10 mmol/L)	22.58	27.31
Cephalothoracic CT	Hemorrhagic focus in the right centrum semiovale and patchy shadows in the lower lobes of both lungs	Not performed
BM cytomorphology	Extremely active proliferation with 96% abnormal promyelocytes	Extremely active proliferation with 91% abnormal promyelocytes
BM Immunophenotyping	Abnormal cells (93.80%) express CD13, CD33, CD64, partially CD117 and do not express CD3, CD5, CD7, CD10, CD11b, CD16, CD34, CD56, HLA-DR.	Abnormal cells (82.93%) express CD13, CD33, partially CD117, CD9, CD123 and do not express CD11b, CD15, CD16, CD34, HLA-DR
BM genetic testing (73 AML-associated gene panel)	PML::RARA*: L-isoform: positive (139%); S-isoform: positive (33.7%); FLT3-ITD: negtive (0%)	PML::RARA* L-isoform: positive (49%); S-isoform: positive (1.42%); FLT3-ITD: positive (54%)
BM cytogenetics	47, XY, +8, t(15; 17) (q24; q21)	47, XY, +8, t(15; 17) (q24; q21)

**Figure 1:**
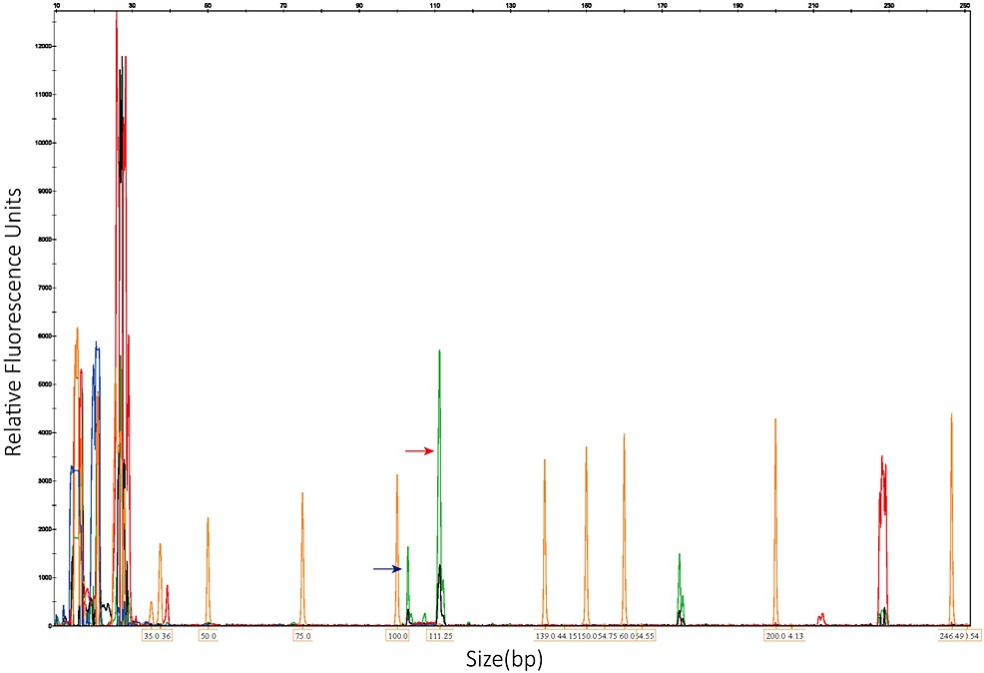
Fluorescence PCR capillary electrophoresis fragment analysis revealed that the PML::RARA fusion transcript was positive for the L-isoform (red arrow, 112 ± 2 bp) and the S-isoform (blue arrow, 102 ± 2 bp). PCR, polymerase chain reaction

**Figure 2:**
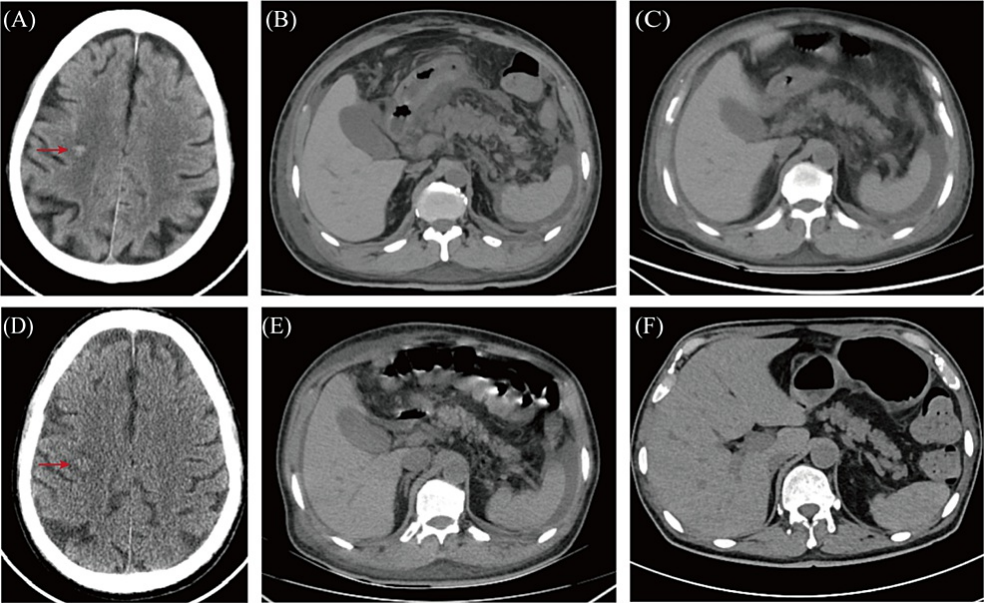
(A) The cranial CT on D1 showed a hemorrhagic focus in the right centrum semiovale; (B) the abdominal CT at night on D8 showed abdominal effusion and subcutaneous exudation; (C) the abdominal CT in the afternoon on D9 showed abdominal effusion, pancreatic edema, and subcutaneous effusion; (D) the cranial CT on D13 showed that the hemorrhagic focus in the right semiovular centrum had become less dense and the peripheral edema had increased; (E) the abdominal CT on D13 showed that the peripancreatic fat space was blurred, and the seroperitoneum and peripancreatic exudate were slightly resorbed; (F) the abdominal CT on D28 showed that the peripancreatic exudate had been resorbed and the seroperitoneum had decreased.

Based on the patient's clinical manifestations and laboratory test results, particularly fluorescent polymerase chain reaction (PCR) capillary electrophoresis fragment analysis and quantitative real-time PCR results indicating positive L- and S-isoform PML::RARA fusion transcripts, the patient was diagnosed with low-risk APL characterized by L- and S-isoform PML::RARA fusion transcripts (Figure 1). This condition was associated with cerebral hemorrhage, pulmonary infection, diabetes mellitus, and renal insufficiency (Figure 2A). Therapeutic intervention for the patient, according to the Chinese guidelines for diagnosis and treatment of APL (2018) [5], included ATRA (25 mg/m^2^/day; 40 mg/day) and ATO (0.16 mg/kg/day; 9 mg/day). Adjuvant therapy consisted of anti-infectives, glycemic control, and blood product transfusions. On D2, the patient rapidly developed generalized swelling, dyspnea, abdominal distension, weight gain of 4.3 kg, and oxygen saturation fluctuating between 82% and 85% after taking only one dose of ATRA (10 mg) on the evening of D1. Therefore, the diagnosis of DS is clear, and the dose of ATRA was reduced to 30 mg/day. The patient was immediately started on idarubicin hydrochloride (10 mg/day, D2, 4, 6) and dexamethasone (20 mg/day). On D4, the dose of ATRA was reduced to 20 mg/day as the WBC continued to rise to 20.60 × 10^9^/L. Subsequently, the patient reported a rapid improvement in dyspnea. Laboratory parameters, including WBC, D-dimer, and FDP, gradually decreased, while FIB returned to normal. However, the patient's abdominal distension persisted despite the above treatment, concomitant albumin infusion, and diuretic therapy with furosemide. On the evening of D8, the patient experienced a sudden onset of abdominal pain. Clinical physical examination revealed tenderness throughout the abdomen but no abdominal rebound pain or tension; peristalsis sounds were noted one to two times per minute; and an abdominal CT scan revealed new bilateral pleural, abdominal, and pelvic effusions as well as subcutaneous exudation in the abdomen and pelvis (Figure 2B). The patient's sudden onset of abdominal pain was initially misdiagnosed as glucocorticoid-induced peptic ulcer disease, and empiric treatment with esomeprazole was initiated. However, the patient reported a significant worsening of abdominal pain and bloating after ten hours. Serum lipase and amylase levels were 1579 U/L (normal range, 23-300 U/L) and 557 U/L (normal range, 35-135 U/L), respectively. Albumin level was 32.4 g/L (normal range, 40-55 g/L), fasting glucose level was 8.79 mmol/L (normal range, 3.90-6.10 mmol/L), calcium ion level was 2.00 mmol/L (normal range, 2.11-2.52 mmol/L), creatinine level was 95.3 μmol/L (normal range, 57-111 μmol/L), and triglyceride level was 2.14 mmol/L (normal range, 0.56-1.70 mmol/L). The thoracoabdominal CT was re-evaluated on the afternoon of D9 and showed increased pulmonary interstitial exudate, bilateral pleural and abodominal effusion, pancreatic edema, subcutaneous effusion, and diffuse thickening of the abdominal and pelvic peritoneum (Figure 2C). These findings led to a diagnosis of acute edematous pancreatitis (AEP), scored as Grade 3 according to Common Terminology Criteria for Adverse Events (CTCAE) v.5.0.

**Figure 3:**
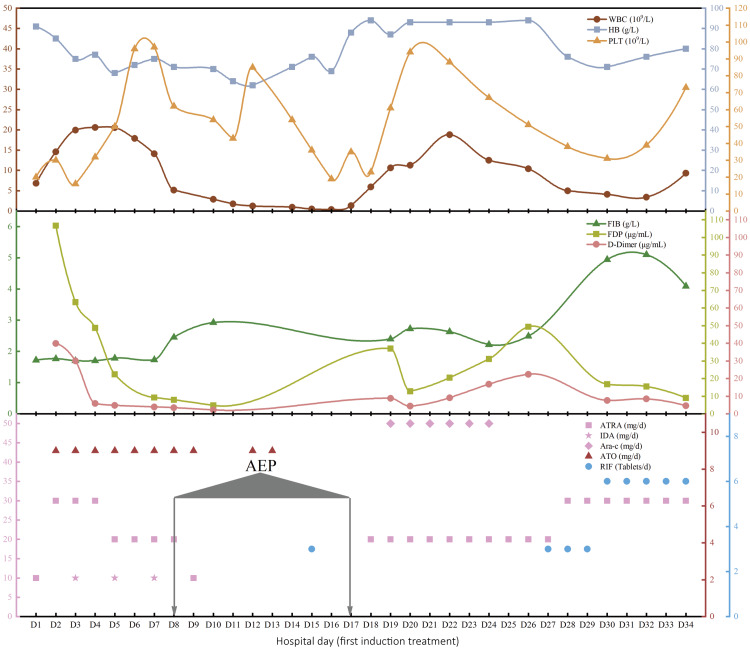
Adjustment of the main therapeutic drug doses and the corresponding dynamic changes in WBC, HGB, PLT, FIB, FDP, and D-dimer during the first induction treatment. Acute edematous pancreatitis (AEP) occurred on D8 to D17. WBC, white blood cell; Hb, hemoglobin; PLT, platelet; FIB, fibrinogen; FDP, fibrinogen degradation products

ATRA and ATO were paused on D9; dexamethasone (20 mg/day) was continued along with food and fluid restriction; intravenous nutritional support; and other therapeutic medications included ulinastatin, esomeprazole, and diuretics. Fortunately, the patient's abdominal distension and pain gradually relieved, and the amylase level decreased to 71 U/L on D12. ATO (9 mg/day) was restarted on the same day, but the patient again developed intolerable abdominal pain and bloating. Repeated cranial and abdominal CT on D13 showed that the hemorrhagic focus in the right centrum semiovale had become less dense and the peripheral edema around the hemorrhagic focus had increased compared to D1 (Figure 2D), the peripancreatic fat space was blurred, the abdominopelvic and peripancreatic exudate was slightly absorbed, and there were bilateral pleural effusions consistent with the findings on D9 (Figure 2E). ATO had to be discontinued again, and Chinese herbal enema, abdominal massage, and acupuncture were used to relieve the patient's abdominal distension until the patient could tolerate D15. On the same day, we tried 75% of the standard dose of RIF (270 mg/tablet; 9 tablets/day) and reduced the dexamethasone to 5 mg/day. Unfortunately, the patient's abdominal distension worsened after only one dose of RIF (three tablets), but gradually improved after discontinuing RIF. We restarted ATRA (20 mg/day) on D18, but the patient experienced chest tightness and shortness of breath the next day, and the WBC increased to 10.63 × 10^9^/L. Cytarabine (50 mg/day) was administered on D19. The patient's leukocytes gradually decreased, and the chest tightness and shortness of breath also gradually improved without stopping ATRA treatment. The patient's abdominal distension remained tolerable during this period. On D27, we again tried low-dose RIF (three tablets per day), and the patient did not experience any worsening of abdominal distension. A repeat thoracoabdominal CT on D28 showed that the patient's bilateral pleural effusions had resolved, the peripancreatic exudate had been absorbed, and the seroperitoneum had decreased (Figure 2F). The doses of ATRA and RIF were gradually increased, as shown in Figure 3, on the premise that the patient could tolerate the abdominal distension. However, when the dose of RIF was increased to 9 tablets per day on D33, the patient experienced a significant worsening of abdominal pain and bloating. As a result, RIF had to be discontinued. Fortunately, the patient's amylase levels were normal, and no abnormal promyelocytes were found in the patient's bone marrow on D34. The patient achieved a CR after the first induction treatment.

Nine months later, the patient experienced a recurrence of buccal mucosal purpura and weakness due to refusal of consolidation and maintenance therapy. The most important test results at the time of relapse are shown in Table 1. It's worth noting that genetic testing at the time of the relapse revealed a positive FLT3-ITD mutation. We used a standard dose of ATRA (40 mg/day) in combination with RIF (12 tablets/day) as a reinduction regimen. However, the patient experienced intolerable abdominal pain and bloating after three days of reinduction therapy. The thoracoabdominal CT scan showed pulmonary exudation and abdominopelvic pleural effusions, as well as subcutaneous exudation, but the amylase level was normal. Based on the experience with the first induction treatment (Figure 3), a substandard but tolerable dose of ATRA (30 mg/day) in combination with RIF (six tablets per day) was selected for the reinduction treatment, and the patient achieved a second CR as no abnormal promyelocytes were found on bone marrow cytomorphology examinations after 28 days of treatment. The patient is currently undergoing consolidation and maintenance therapy according to Chinese guidelines. However, due to the patient's indelible fear of abdominal distension and pain, any treatment still requires constant explanation and persuasion.

## Discussion

APL diagnosis based on the cytogenetic finding of t(15;17)(q24;q21) and/or the detection of the PML::RARA fusion transcript. The 5th edition of the World Health Organization Classification of Hemolymphoid Tumors explicitly defined APL as *APL with PML::RARA fusion* to distinguish it from other variants of *APL* that lack the PML::RARA fusion transcript but possess the morphologic and immunophenotypic features of typical APL [6]. The RARA gene is located at 17q21 and contains 10 exons, with only one breakpoint in intron 2. The PML gene is located at 15q24 and contains nine exons with breakpoints in intron 6, exon 6, and intron 3, commonly known as breakpoint cluster region (bcr) 1, bcr2, and bcr3, respectively. Three different isoforms can be generated depending on the breakpoint used and subsequent splicing, as mentioned earlier. Notably, the incidence of the three PML::RARA sub-isoforms varies with geographic location and ethnicity. However, other fusion transcripts involving the RARA gene are relatively rare and mostly reported as single cases or case series [1,7]. Therefore, it is necessary to further categorically summarize their clinical features, treatment response, and prognosis. In this case, both L- and S-isoform fusion transcripts are present, but cytomorphology and immunophenotyping did not identify two distinct groups of abnormal promyelocytes. The sub-isoforms of PML::RARA may have little effect on the cytomorphologic and immunophenotypic features of abnormal promyelocytes in APL. We found that similar reports are rare in the literature, so we report this case for further collection to retrospectively analyze the unique features of the mixed isoforms of APL. The patient in this case initially had a WBC less than 10 × 10^9^/L and was stratified as low risk, but demonstrated rapid onset of DS and poor tolerance to ATRA, ATO, and RIF. However, the duration of induction treatment required to achieve CR was not significantly prolonged despite receiving substandard doses of ATRA, ATO, or RIF. In addition, the patient acquired FLT3-ITD mutations at relapse compared to the initial diagnosis. However, it is unclear whether the features observed in this patient are unique to this mixed isoform of APL or are individual to this patient.

The pathogenesis of DS is complex and not fully understood. It is mainly a combination of factors, including a systemic inflammatory state, increased vascular permeability, endothelial damage, and increased adhesion of tumor cells to each other or the endothelium, caused by the release of pro-inflammatory cytokines such as interleukin 1 beta (IL1B), interleukin 6 (IL6), cathepsin G, interleukin 8 (IL8), and tumor necrosis factor (TNF), as well as increased expression of lymphocyte function-associated antigen 1 (LFA1), intercellular adhesion molecule 2 (ICAM2), and very late antigen 4 (VLA4). The development of DS varies depending on several factors, such as risk stratification and therapeutic regimen. Severe DS occurring within the first week is associated with a higher mortality rate compared to the second week and beyond [3,8]. This patient experienced a rapid rise in WBC on the second day of induction treatment with ATRA in combination with ATO and developed chest tightness, shortness of breath, abdominal distension, generalized swelling, and weight gain, meeting the diagnostic criteria for severe DS. It appears that the patient's severe abdominal distension was related to the doses of ATRA, ATO, and RIF, perhaps just a dose-dependent toxic side effect of these drugs that cannot be considered a clinical feature of DS, but it was the reason for the tortuous course of the initial induction treatment, as we had to make repeated attempts to ensure the patient's maximum tolerated doses (Figure 3). This may suggest that patients with higher vulnerability to treatment toxicities may be allowed to continue receiving standard doses of these drugs if they survive early treatment-related mortality by reducing the drug dose. However, a small cohort analysis of elderly patients confirmed that suboptimal induction treatment with modified or reduced personalized approaches may lead to dismal outcomes [9]. Therefore, drugs such as venetoclax, a selective inhibitor of B-cell lymphoma 2 (BCL-2), which has been used successfully in the treatment of elderly patients with acute myeloid leukemia, may be worthy of further investigation.

The possible causes of AP during APL treatment have been summarized as adverse drug reactions to ATRA or ATO, hypercalcemia, hypertriglyceridemia, concomitant sepsis, disseminated intravascular coagulation, and pancreatic edema due to DS [10]. In this case, the patient did not have hypercalcemia, hypertriglyceridemia, or concomitant sepsis. Multiple CT scans showed pleural, abdominal, and pelvic effusions, and edema was observed in abdominal organs such as the pancreas and intestines (Figure 2). We thought that the pancreatic edema caused by DS was responsible for the AP in this patient. Therefore, more attention should be paid to the identification of AP when APL occurs with acute abdominal pain, and appropriate treatment should focus on resolving the causes of AP.

## Conclusions

APL may have a mixed L- and S-isoform PML::RARA fusion transcript and may acquire the FLT3-ITD mutation in relapse. AEP is a rare manifestation of DS and should be recognized when APL is associated with acute abdominal pain. ATRA, ATO, and RIF may cause dose-dependent abdominal distension, for which a dose reduction of these drugs may be an option, but the impact on long-term survival is uncertain.
